# Shooting the messenger: a systematic review investigating extracellular vesicle isolation and characterisation methods and their influence on understanding extracellular vesicles-radiotherapy interactions in glioblastoma

**DOI:** 10.1186/s12885-023-11437-6

**Published:** 2023-10-05

**Authors:** Stephen David Robinson, Mark Samuels, William Jones, Duncan Gilbert, Giles Critchley, Georgios Giamas

**Affiliations:** 1 Department of Biochemistry and Biomedicine, School of Life Sciences, University of Sussex, John Maynard Smith Building, Falmer, Brighton, BN1 9QG UK (SDR, MS, WJ, GG); 2 Sussex Cancer Centre, University Hospitals Sussex NHS Foundation Trust, Brighton, UK (SDR, DG); 3 Medical Research Council Clinical Trials Unit, University College London, London, UK (DG); 4 Department of Neurosurgery, University Hospitals Sussex NHS Foundation Trust, Brighton, UK (GC)

**Keywords:** Glioblastoma, Radiotherapy, Extracellular vesicles

## Abstract

**Background:**

Extracellular vesicles (EVs) hold promise for improving our understanding of radiotherapy response in glioblastoma due to their role in intercellular communication within the tumour microenvironment (TME). However, methodologies to study EVs are evolving with significant variation within the EV research community.

**Methods:**

We conducted a systematic review to critically appraise EV isolation and characterisation methodologies and how this influences our understanding of the findings from studies investigating radiotherapy and EV interactions in glioblastoma. 246 articles published up to 24/07/2023 from PubMed and Web of Science were identified using search parameters related to radiotherapy, EVs, and glioblastoma. Two reviewers evaluated study eligibility and abstracted data.

**Results:**

In 26 articles eligible for inclusion (16 investigating the effects of radiotherapy on EVs, five investigating the effect of EVs on radiation response, and five clinical studies), significant heterogeneity and frequent omission of key characterisation steps was identified, reducing confidence that the results are related to EVs and their cargo as opposed to co-isolated bioactive molecules. However, the results are able to clearly identify interactions between EVs and radiotherapy bi-directionally within different cell types within the glioblastoma TME. These interactions facilitate transferable radioresistance and oncogenic signalling, highlighting that EVs are an important component in the variability of glioblastoma radiotherapy response.

**Conclusions:**

Future multi-directional investigations interrogating the whole TME are required to improve subsequent clinical translation, and all studies should incorporate up to date controls and reporting requirements to increase the validity of their findings. This would be facilitated by increased collaboration between less experienced and more experienced EV research groups.

## Importance of the study

Radiotherapy is one of the most effective treatments for glioblastoma, however we still cannot predict which patients will respond to treatment. Extracellular vesicles hold significant promise for improving understanding of intercellular communication within the tumour microenvironment following radiation in glioblastoma, as well as for their development as a blood based liquid biopsy. We have systematically reviewed the literature and highlighted significant variability in the robustness of techniques used to isolate and characterise extracellular vesicles which significantly impacts the ability to pull together a coherent narrative of how radiotherapy and extracellular vesicles interact in glioblastoma. This review highlights the need for reproducible and transparent protocols, alongside clear and descriptive reporting, to enable the full benefits of this technique to be realised and to facilitate their potential development as a minimally invasive blood-based biomarker.

## Introduction

Extracellular vesicles (EVs) are small, lipid bilayer enclosed particles containing bioactive materials including proteins, lipids, nucleic acids, and metabolites. Among many proposed functions are their potential to contribute to intercellular signalling by transferring their bioactive cargo [1]. Cancer cell-derived EVs are associated with many cancer hallmarks [2], implicated in establishing and developing the tumour microenvironment (TME) through facilitating bidirectional oncogenic signalling with surrounding non-malignant cells [3], and identified as a mechanism of treatment resistance [4]. Extracellular vesicles require active secretion from living cells, whilst their lipid bilayer membrane protects their cargo from degradation. These features, alongside their short half-life, their ability to reflect their cell of origin, and their presence in the peripheral circulation, raise the possibility that they could be used as a dynamic liquid biopsy biomarker for various tumour types.

Glioblastoma (GBM) is characterised by intratumoural heterogeneity and a complex TME, which both drive treatment resistance and a dismal prognosis of 14.6 months [5]. Radiotherapy remains one of the most effective treatments for GBM [6, 7], however it is currently unknown why individuals have varying responses to radiation. It is now known that the different components of the TME can drive radiation response [8], whilst recent understanding has implicated intercellular communications by EVs within the TME as underpinning this radiotherapy resistance [9]. Additionally, post-radiotherapy an inflammatory pseudoprogression can develop complicating MRI-based surveillance [10]. Therefore, understanding EV-radiotherapy interactions in GBM and the development of an EV based liquid-biopsy which informs on the underlying biological processes within the GBM tumour, are important overlapping areas of research.

EVs can be isolated from biofluids using several techniques based on their physical characteristics (size or density) or their composition (surface markers). Unfortunately, there is no consensus on the optimal EV isolation technique. Different techniques result in differences in yield, purity, and suitability for downstream applications. Additionally, without appropriate characterisation, it is difficult to confirm whether identified findings are related to EVs cargo, biomolecules captured at the EV surface, or a contaminant that has co-separated during isolation [11]. To improve the quality and reproducibility of EV research, the International Society for Extracellular Vesicles (ISEV) released updated guidelines on the minimal information for studies of EVs in 2018 (MISEV2018) and recommended the standardised terminology of EVs as opposed to exosome, microvesicle or microsome unless biogenesis had been confirmed [12].

**Fig. 1:**
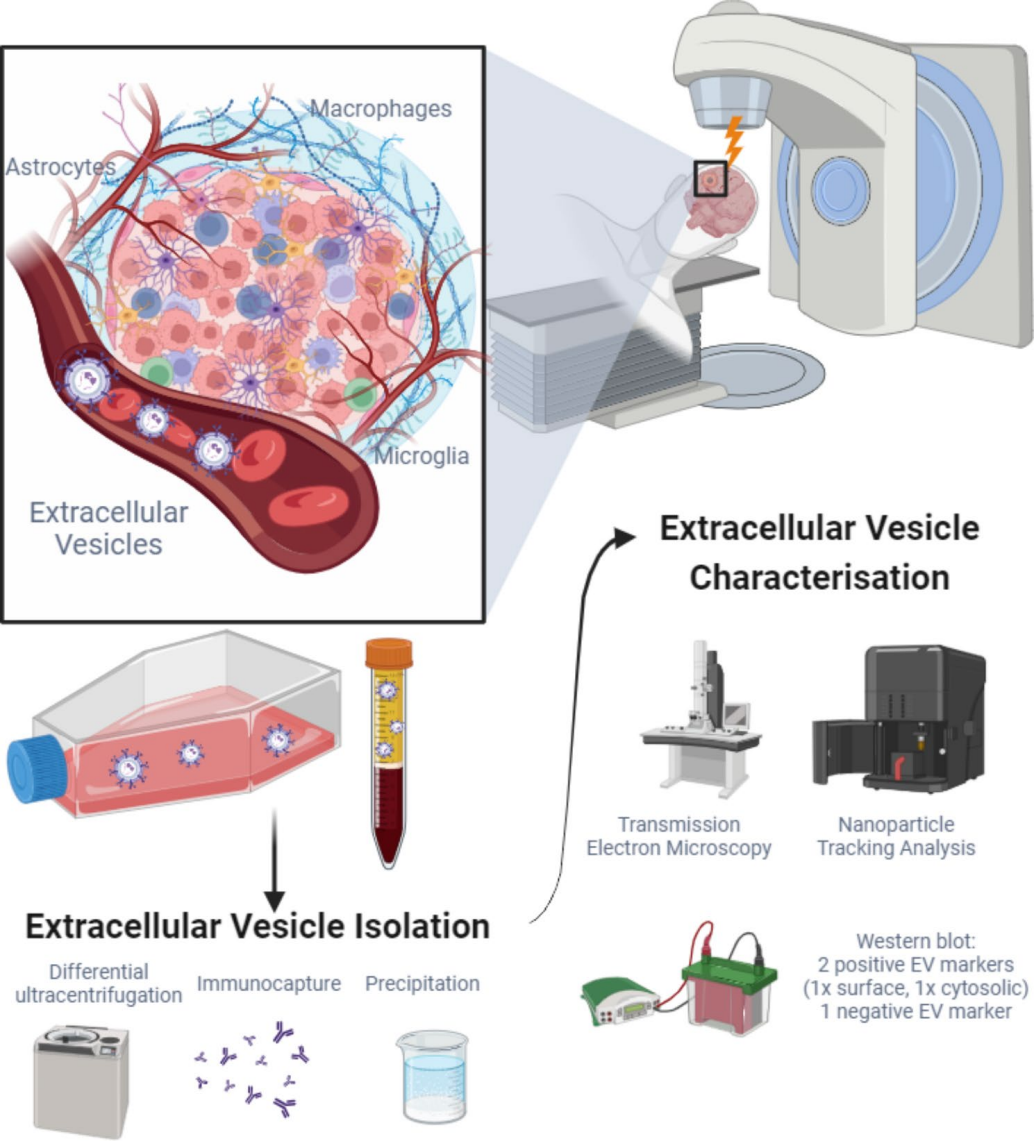
Graphical overview of the systematic review

In this systematic review, we report on the quality of the EV enrichment and validation techniques, plus how this affects our interpretation, of the studies investigating interactions between radiotherapy and EVs in GBM, summarised in Fig. 1.

## Materials and methods

This systematic review was guided by the Preferred Reporting Items for Systematic Review and Meta-Analyses (PRISMA) guidelines [13].

### Literature search

Web of Science and PubMed were searched on the 24/07/2023 to identify all studies published up to that date using the following search terms: (“extracellular vesicles” OR EVs OR microsomes OR microvesicles OR exosomes) AND (radiotherapy OR radiation) AND (GBM OR glioblastoma or glioma).

### Inclusion/exclusion criteria

We aimed to identify all articles (full-text or abstract) investigating interactions between radiation and EVs in GBM. Given the bi-directional communication of EVs within the TME, studies were eligible for inclusion if they either: investigated changes in EVs following radiotherapy of GBM (cell lines or clinically) or if they co-cultured GBM cells with EVs and assessed subsequent radiosensitivity.

Articles were excluded based on the following criteria: [1] duplicates (n = 61), [2] written in a non-English language (n = 3), [3] retracted following publication (n = 1), [4] review articles (n = 78), [5] not investigating the interaction between radiotherapy and EVs (n = 72), and [6] conference abstracts with a subsequent full text publication (n = 5).

All titles and abstracts were screened by two authors (SDR and MS) against this inclusion/exclusion criteria. The full-texts of potentially eligible articles were reviewed to confirm eligibility and the reference lists of included articles and relevant excluded review articles were examined to ensure all relevant articles were included. Any uncertainties regarding article inclusion were resolved through discussion by the authors (SDR, MS and WJ).

### Data collection

Data extraction was performed by two authors (SDR and MS) following a thorough review of each article according to criteria agreed by the authors in advance. Relevant criteria included: author, publication year, cell line and/or patient population studied, radiotherapy details, EV isolation methods, EV characterisation methods and results.

### Study evaluation

One of the main risks with EV research is assigning identified biological effects to EVs and their cargo as opposed to co-isolated biomolecules. An assessment of the strength of the biological findings has been made in relation to the robustness and appropriateness of that studies EV methodology as recommended in the MISEV2018 criteria.

### Synthesis of methods

Results were categorised under the following headings: effect of radiotherapy on EVs, effect of EVs on radiotherapy response, clinical studies. Within categories, studies were grouped based on the non-GBM cell included in the investigation. The proportion of studies within categories using different isolation/characterisation methods was quantified and related to the risk of bias assessment and overall quality of the EV methodology.

## Results

### Literature search and study selection

**Fig. 2:**
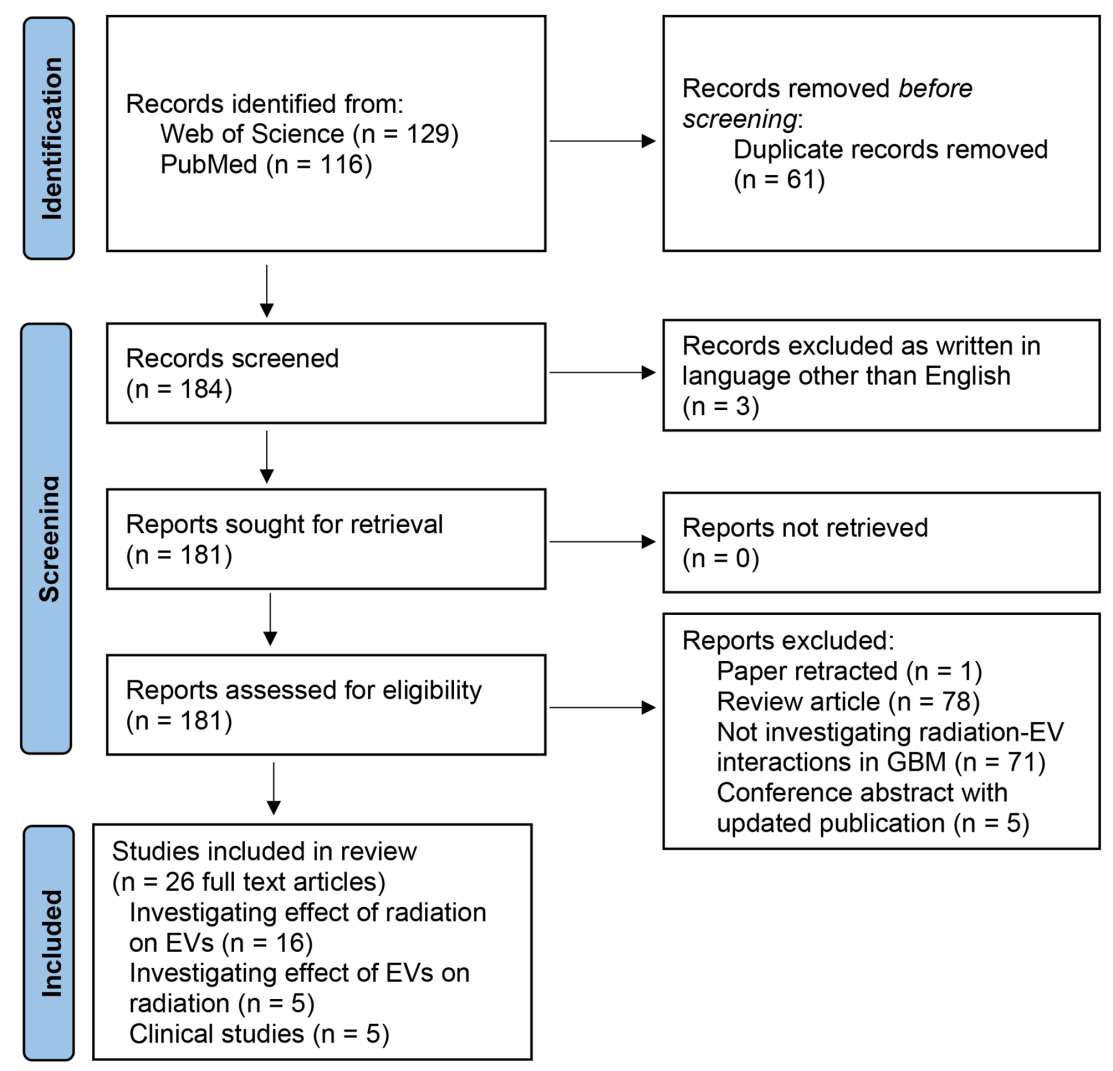
The PRISMA flow diagram of study selection

245 articles were identified, and after duplicate removal, 184 articles were screened, and 158 articles were excluded according to the eligibility criteria. A total of 26 articles were included. The PRISMA schematic is demonstrated in Fig. 2.

Included studies were published between 2013 and 2023, used heterogeneous radiotherapy protocols, three EV isolation methods, and varying EV characterisation techniques. Within the three subcategories, 16 studies investigated the effect of radiotherapy on glioma-derived EVs (Table 1), five investigated GBM response to radiation following EV co-culture (Table 2), and five clinical studies investigated EVs in GBM patients at timepoints around their radiotherapy (Table 3).

### EV isolation methodology

Four methods for EV isolation are used: immunocapture (used in 1/16, 0/5 and 3/5 studies in each category), differential ultracentrifugation (used in 9/16, 3/5 and 0/5 studies), precipitation (used in 6/16, 2/5 and 1/5 studies) and size exclusion chromatography (used in 0/16, 0/5 and 1/5 studies).

Immunocapture uses antibodies to target specific EV surface markers to selectively bind to EVs. It isolates a more homogenous EV population; however, the yield is lower, and this technique may be less suitable for discovery research as it enriches for a sub-population of all EVs due to the absence of a universal EV antigen.

Ultracentrifugation has typically been the most frequently used method for EV isolation; however, it is time-consuming, technically challenging, and imparts significant mechanical stress to the EVs during isolation. Additionally, multiple variables influence the population of EVs isolated and the sample purity [11] including the number, speed, and length of centrifugation spins, and the use of filtration or other methods during enrichment.

Precipitation is a quicker, simpler, and more standardised process typically using commercial kits. However, it is non-targeted, resulting in isolation of the majority of EVs, but at the expense of a greater concentration of contaminants than other methods.

Size exclusion chromatography is emerging as one of the most frequently utilised EV isolation methods. It uses size-based separation to isolate EVs, is much quicker and simpler than differential ultracentrifugation, and should have less co-isolated products than precipitation based-methods with the main non-EV component large protein complexes and lipoproteins.

### EV characterisation methodology

Following isolation of the putative EV fraction, the MISEV 2018 guidelines recommend multiple characterisation steps to confirm changes are EV-related and not due to co-isolated molecules. Two complementary techniques are recommended for single EV analysis, including high resolution microscopy (e.g. transmission electron microscopy [TEM]) complemented by non-high resolution imaging techniques (e.g. nanoparticle tracking analysis [NTA]), in addition to the enrichment of positive (one membrane and one cytosolic) EV markers and the depletion of negative markers (frequent contaminants such as albumin and/or cellular components not expected in EVs). Moreover, to clarify that identified changes are related to EV cargo, the addition of detergents to lyse the cell membrane and proteinases to digest the bioactive cargo can be used, with loss of the observed activity expected with pre-treatment using both proteinase and detergent.

#### Studies investigating the effect of radiotherapy on EVs

For the studies investigating how radiotherapy affects glioma-derived EVs, three investigated the effect of radiation on EV numbers or on EV composition to understand radioresistance, seven studied the effects of radiotherapy-induced EVs on glioma cells, five studied the effects of radiotherapy-induced EVs on cells within the TME, and one studied the effects of radiotherapy-induced EVs on skeletal muscle.

**Table 1:** A summary of studies investigating the effect of radiotherapy on EVs in GBM.

Author	Year	Radiotherapy details	Donor cell	EV isolation	EV confirmation	Recipient cell	Results
Jennrich [14]	2022	Modality: Cabinet xray irradiation, 230 MeV cyclotron - Low LET or high LET proton irradiation. Treatment: 0, 2, 4, 5, 6 and 10 Gy in 1 fraction.	4 GBM cell lines (A172, LN229, U373, T98G).	72-hour conditioned media. Centrifugation (500 g 5 min, 2000 g 15 min) 0.22 μm filtering. Supernatant incubated with fluorescent antibodies.	Imaging flow cytometry (CD9, CD81) to assess concentration.	NA	Increases in EVs following radiation.
Ramakrishnan [15]	2020	Modality: No details. Treatment: 0 and 6 Gy in 1 fraction in cells. 0 and 6 Gy in 2 fractions in subcutaneous mouse model. 0 and 10 Gy in 5 fractions in intracranial mouse model.	1 GBM cell line (LN340) and 1 primary GBM culture cells (BT-83).	? hour conditioned media. Total Exosome Isolation Reagent or ExoQuick-TC ULTRA EV isolation kit.	Nanosight LM-10 analysis to assess size and concentration. Western blot of EV (CD9, CD81, TSG101, HSC70) and purity control (ApoA1, alpha tubulin) markers. Recipient cell uptake using PKH67 fluorescent labelling. Repeat experiments using RNAse +/- proteinase K +/- detergent digested EVs.	NA	Reduction in EVs following radiation. Reduced donor cell miR-603 through EV trafficking leading to radioresistance.
Whitehead [17]	2023	Modality: No details Treatment: 0 and 2 Gy in 1 fraction	2 GBM cell lines (U87MG, LN229) and 2 primary GBM culture cells (MU4, MU41).	24-hour conditioned media. Differential ultracentrifugation: 300 g 10 min, 2000 g 15 min, 10,000 g 30 min, 100,000 g 60 min, washed with PBS and 100,000 g 60 min.	Nanosight 300 analysis and Cryo-electron microscopy to assess size and concentration. Western blot for EV (ALIX), purity control (calnexin, tubulin) markers. Recipient cell uptake using DiI fluorescent labelling and dye-only control.	NA	Increase in EV number following radiation but not size. Upregulation of EV proteins associated with invadopodia and TME interactions.
Arscott [18]	2013	Modality: Cabinet xray irradiation. Treatment: 0 and 4 Gy in 1 fraction. 1 line also received 0, 2, 4, 6 and 8 Gy in 1 fraction.	3 GBM cell lines (LN18, U87, U251) and 2 GSC lines (GBAM1, GBJM1).	12-48-hour conditioned media (mostly 24 h). Differential ultracentrifugation: 200 g 5 min, 3000 g 15 min, 110,000 g 120 min, washed with PBS and 110,000 g 120 min.	Transmission electron microscopy and Nanosight LM10 analysis to assess size and concentration. Western blot for EV (CD9, TSG101, ALIX), purity control (Actin) and loading control (Ponceau S) markers. Recipient cell uptake using PKH26 fluorescent labelling. Repeat experiments using Tx-100 digested EVs.	1 GBM cell line (U87).	Radiation increases EV number but not size. Radiation-derived EVs leads to recipient GBM cell migration.
Baulch [22]	2016	Modality: Caesium gamma irradiation. Treatment: 0, 1, 2, and 5 Gy in 1 fraction 2.5 and 5 Gy in 5 fractions (5 days) 5 and 10 Gy in 10 fractions (12 days) 10 Gy in 20 fractions (26 days).	1 primary GBM cultures (301).	96-hour conditioned media. Centrifugation (3000 g 15 min) followed by ExoQuick TC EV isolation kit.	Nanosight 300 analysis to assess size and concentration.	2 primary GBM cultures (381, 408).	Multi-fraction radiotherapy leads to radioresistance and increased MMP2 levels.
Mrowczynski [23]	2018	Modality: No details. Treatment: 0, 3 or 12 Gy in 1 fraction.	1 GBM cell line (U87) and 2 non-GBM cell lines.	48-hour conditioned media. Centrifugation (3000 g 15 min) followed by ExoQuick TC EV isolation kit.	Transmission electron microscopy and nanoparticle tracking analysis (Zetasizer and Nanosight300) to assess size and concentration. Western blot for EV markers (CD81, TSG101). Recipient cell uptake using PKH67 fluorescent labelling.	1 GBM cell line (U87) and 2 non-GBM cell lines.	Radiotherapy increases EV concentration. Radiotherapy induced EVs lead to radioresistance when co-cultured with glioma cells due to changes in their miRNA, RNA and protein cargo.
Pavlyukov [19]	2019	Modality: No details. Treatment: 0, 6, and 12 Gy in 1 fraction.	Multiple fresh primary GBM cultures.	? hour conditioned media Centrifugation (1000 g 10 min) then 0.8 μm filtering. Samples also underwent ultracentrifugation: 16,500 g 20 min OR 120,000 g 70 min.	Nanosight 500 analysis to assess size and concentration. Recipient cell uptake using PKH26 fluorescent labelling.	Multiple fresh primary GBM cultures and fresh primary GBM cultures in NOD scid mouse xenograft model.	Radiation increases EV concentration. Co-culture leads to radioresistance, temozolomide resistance, proliferation, and invasion.
Pineda [20]	2019	Modality: Photon irradiation via linear accelerator. Treatment: 50 Gy in 1 fraction.	1 GBM cell line (C6).	72-hour conditioned media. Centrifugation (200 g ?mins, 14,000 g 20 min).	Transmission electron microscopy and nanoparticle tracking analysis to assess size and concentration. Flow cytometry of EV marker only (Annexin V).	Wistar rat sub-cutaneous xenograft model.	Radiotherapy increases EV concentration. Radiotherapy-induced EVs cause immune mediated tumour shrinkage.
Zhao [21]	2019	Modality: Cobalt gamma irradiation. Treatment: 60 Gy in 12 fractions to develop radioresistant line. 5 Gy in 1 fraction.	1 GBM cell line (U251) with control and radioresistant lines.	? hour conditioned media Differential ultracentrifugation: 550 g 15 min, 16,500 g 30 min, 0.22 μm filtering, 100,000 g 120 min, washed with PBS and 100,000 g 120 min.	Transmission electron microscopy and Nanoparticle tracking analysis using Zetaview to assess size and concentration. Recipient cell uptake using PKH67 fluorescent labelling.	1 GBM cell line (U251).	EVs provide transferrable radioresistance through the transfer of circRNA and miRNA linked to cell cycle and p53 signalling pathways.
Wang [24]	2021	Modality: Photon irradiation via linear accelerator. Treatment: 0, 2, 4, 6, 8 and 10 Gy in 1 fraction for cells. 0 and 10 Gy in mice.	2 GBM cell lines (SW1783, U118).	48-hour conditioned media. Centrifugation (1000 g 10 min) followed by Total Exosome Isolation Reagent.	Transmission electron microscopy and Nanosight 300 analysis to assess size and concentration. Western blot of EV markers (CD63, TSG101) and purity control (GAPDH) markers.	2 GBM cell lines (SW1783, U118) and BABL/c xenograft mouse model.	
Colangelo [26]	2020	Modality: Caesium gamma irradiation. Treatment: 0, 2, 4, 6, 8 and 10 Gy in 1 fraction	3 GBM cell lines (T98, U87, U118).	24-, 48- or 72-hour conditioned media (new media every 24 h). Differential ultracentrifugation: 2000 g 10 min, 10,000 g 30 min, 100,000 g 60 min, washed with 0.22 μm filtered PBS, and 100,000 g 90 min.	Cryo-electron microscopy and LM10 nanoparticle tracking analysis for size and concentration. Western blot for EV (CD63, TSG101, ALIX), purity control (GM130) and loading control (Ponceau S) markers. Recipient cell uptake using PKH67 fluorescent labelling.	1 astrocytoma cell line (SVG p12).	Radiotherapy increased EV CD147 levels at 24/48 h which resolved by 72 h. Co-culture with astrocytes led to increases in MMP9 secretion.
Briand [28]	2020	Modality: No details for cell line irradiation. Small animal xray irradiator. Treatment: 0 or 10 Gy in 1 fraction in cells. 0 or 20 Gy in 10 fractions (10 days) in mice.	1 GBM cell line (U87).	24-hour conditioned media. ExoQuick TC ULTRA EV isolation kit directly from 5ml media. Direct EV miRNA extraction using exoRNeasy Serum Plasma Kit.	No clear confirmation.	1 Natural Killer Cell cell line (NK92) and PC3 mouse xenograft model.	Radiotherapy increases EV miR-378a-3p which leads to reduced NK cell cytotoxicity through reduced granzyme B levels.
Yang [27]	2021	Modality: Cabinet xray irradiation. Treatment: 0 and 10 Gy in 1 fraction	1 mouse glioma cell line (GL261).	3-hour conditioned media. Centrifugation (2000 g 30 min) followed by Total Exosome Isolation Reagent.	Zetasizer Nano ZS90 to assess size and concentration. Recipient cell uptake using DiI fluorescent labelling.	Freshly culture neural stem cell culture and healthy C57BL/6 mice.	Radiotherapy reduces neural stem cell proliferation with subsequent neurocognitive effects.
Tian [29]	2022	Modality: No details for cell line or mouse irradiation. Treatment: 0, 2, 4 and 8 Gy in 1 fraction in cells. No details in mice.	1 mouse glioma cell line (GL261) and 2 GBM cell lines (LN229, LN308).	48-hour conditioned media. Differential ultracentrifugation: 300 g ? mins, 2000 g ? mins, 10,000 g ? mins and 100,000 g 180 min.	Nanosight 300 analysis of whole and iodixanol density-gradient centrifugation fractions to assess size and concentration. Western blot of EV (CD63, CD81, ALIX, TSG101) and purity control (Cox IV, calreticulin, Bip) markers.	Healthy volunteer T cells (from peripheral blood mono-nuclear cells separated using anti CD3 beads).	Radiotherapy increases FoxP3 + T cells and decreases Th1 cells via B7-H4.
Zhang [30]	2022	Modality: Photon irradiation via linear accelerator. Treatment: 0 and 2 Gy in 1 fraction. 0 or 6 Gy in 1 fraction in zebrafish.	2 GBM cell lines (U87, U251).	? hour conditioned media. Differential ultracentrifugation: 300 g 10 min, 2000 g 10 min, 10,000 g 10 min and 110,000 g 120 min.	Transmission electron microscopy and Nanosight 300 analysis to assess size and concentration.	1 microglia cell line (HMC3).	Radiotherapy induced microglial M2 polarisation resulting in associated GBM cell proliferation.
Shin [31]	2022	Modality: Cabinet xray irradiation. Treatment: 0 and 6 Gy in 1 fraction in cells. 0 or 10 Gy in 5 fractions in mice.	1 GBM cell line (U87).	48-hour conditioned media. Differential ultracentrifugation: 0.45 μm filtering, 300 g 10 min, 1000 g 20 min, 10,000 g 30 min, 0.22 μm filtering and 100,000 g 60 min.	Transmission electron microscopy to assess size. Western blot for EV markers only (CD9, CD63, CD81). Recipient cell uptake using DiO fluorescent labelling.	1 myoblast cell line (C2C12) and 1 myotube cell line (C2C12).	Radiotherapy induced EVs led to a muscle cachexia phenotype.

### Radiotherapy-induced EVs

Jennrich et al. [14] compared the difference between proton- and photon-radiotherapy on glioma cell survival. A range of single fraction radiation doses and multiple GBM cell lines were investigated. They noticed a dose dependent increase in EV secretion from two of their four cell lines. Given that these two cell lines were most sensitive to radiation induced cell death, they attributed this finding to differing levels of apoptosis.

They used direct fluorophore antibody tagging of CD9 and CD81 (EV-associated surface markers) in 72-hour conditioned media followed by flow cytometry to measure EV levels following sham or real irradiation. However, flow cytometry assessment of CD9/81 particles is their only form of EV characterisation.

This study primarily focusses on EV counts following radiotherapy, and as such uses appropriate measurement techniques. Due to this focus and lack of further analysis minimal biological information regarding mechanisms of EV increase can be identified from these results.

To investigate why radiation (6 Gy) impacted miR-603 levels, Ramakrishnan et al. [15] studied the role of EVs as a mechanistic link in this process. They identified reduced EV concentration following radiation, in contrast to other studies, but found that EV miR-603 concentration increased. They subsequently demonstrated that treatment with a standard small EV release inhibitor (GW4869) abrogated the reduction of miR-603, suggesting that cells secrete miR-603 via EVs as a mechanism to reduce intracellular miR-603 following radiation. Secretion of miR-603 leads to the development of a stem cell like state leading to radioresistance.

They used a single fraction radiation dose and isolated EVs through precipitation, although it is unclear at which timepoint post-radiation they collected media for EV isolation. They performed NTA alone for single EV analysis but performed Western blots of multiple positive and negative EV markers. Additionally, they repeated the functional experiments using RNAse +/- proteinase K +/- detergent to digest EVs to confirm the effects were related to EV cargo. They also utilised fluorescent tagging of EVs to try to confirm recipient cell uptake. However, whilst they did include a control experiment which did not add the EVs or fluorescent label, they did not include an EV only control or a dye only control (adding the fluorescent label but not the EVs) so it is uncertain if the identified fluorescent particles are EVs, labelled co-isolated molecules or micelles of the lipophilic tracer [16].

Despite the unknown timescale for EV collection post-radiation, which is known to influence EV cargo changes following radiotherapy, the substantial functional characterisation suggests that the identified changes in miR-603 levels are related to EV secretion.

Finally, by investigating high and low invadopodia glioma cell lines, Whitehead et al. [17] identified differential proteomes in EVs cargo, demonstrated that co-culture of high invadopodia-derived EVs with low invadopodia cells increased MMP2 secretion and invadopodia, and showed miRNA changes in recipient cells associated with invadopodia. They subsequently investigated the effect of radiotherapy (2 Gy) plus temozolomide on EV cargo, identifying differentially expressed proteins associated with invadopodia and TME interactions. Finally, they demonstrated increased EV number following radiation, but no change in size, which was increased further when combining radiation with temozolomide.

They performed differential ultracentrifugation, involving slower spins to remove cells and cell debris, faster spins to remove large EVs, and then very fast spins (typically ≥ 100,000 g) to pellet the EV fraction. They also performed a washing step, shown to reduce the protein contamination, followed by a final very fast spin to pellet the isolated EV fraction. They performed a robust characterisation protocol, using cryo-electron microscopy and NTA for single EV analysis, and Western blot analysis of a single EV marker plus purity control markers. Additionally, they used fluorescent labelling to demonstrate recipient cell uptake and included a dye only experiment as a control.

This was a well performed study, and the only paper to include a dye-only control with their fluorescent labelling experiment. Despite not being the primary focus, they were able to demonstrate that radiation altered EV secretion and content. There is a suggestion that these changes would lead to increased invasiveness in recipient cells, having demonstrated increased invasiveness following radiation treatment and EV co-culture individually, although this has not been specifically investigated.

### Radiotherapy-induced EVs effects on glioma cells

Seven studies assessed radiotherapy-induced EVs effects on glioma cells. Four utilised ultracentrifugation to isolate EVs and three used precipitation, with each group discussed separately.

Arscott et al. [18] isolated EVs from GBM, glioma stem cell [GSC] and astrocyte cell lines following one single fraction dose of radiation (4 Gy). Additionally, they performed a radiotherapy dose-response assessment and a conditioned media collection timepoint assessment in a single cell line. They demonstrated an increased production of EVs following radiotherapy, but no change in mean size, to suggest that the increase in EVs was unlikely to be due to increasing apoptotic body (a form of large EV) secretion. They identified that co-culture of radiation-induced EVs with GBM cells leads to increased migration alongside mRNA changes associated with cell migration.

They performed differential ultracentrifugation to isolate EVs, albeit without a spin to remove larger EV and cell debris components. Robust characterisation was performed using TEM and NTA for single EV analysis, Western blot analysis of several known EV and purity control markers. They also performed fluorescent uptake, although without an EV only or dye only control, and detergent-treated experiments to verify EV uptake as the likely cause of these changes.

This is one of the most robust characterisation protocol of all identified studies. The level and depth of their characterisation provides clear evidence to justify their conclusion that radiotherapy of GBM cells can lead to increased migration of the surrounding tumour cells.

To investigate the effect of GBM cell apoptosis, primarily induced by radiotherapy, on other GBM cells within the TME, Pavlyukov et al. [19] performed co-culture of GBM cells with radiation-induced EVs. They demonstrated an increase in EV number after radiation alongside increased proliferation, migration, invasion, and resistance to temozolomide and radiation. Proteomic analysis identified spliceosome changes including that RBM11 (a mesenchymal splicing factor upregulated in proneural subtype donor cells following radiation) is exported in EVs in a caspase-dependant manner following radiation leading to endogenous upregulation of RBM11 in the recipient cells.

They used a reasonable differential ultracentrifugation protocol. However, it is unclear how long their media was conditioned for before isolation. They performed NTA and fluorescent NTA for single vesicle characterisation, and performed Western blots of multiple positive EV markers but there was no assessment of common co-isolated proteins.

This was a complicated study not primarily focussed on understanding the effects of radiation on EVs, but used radiotherapy as a method to cause apoptosis which was their focus. With this focus in mind, the overarching conclusion that radiotherapy induced apoptosis leads to a more aggressive phenotype in recipient cells is supported by these results.

By investigating an extremely high radiation dose (50 Gy), Pineda et al. [20] demonstrated an increase in EV number, alongside global protein content changes as assessed by gel electrophoresis. Injection of their isolated EV containing fraction into a subcutaneous glioma rat model led to an immune-mediated tumour reduction through apoptosis, an increase in tumour infiltrating CD4/CD8 lymphocytes, no change in blood/spleen lymphocytes and few other immune cell alterations.

They used an unusual differential ultracentrifugation protocol, performing only a slower spin to remove cells and a final centrifugation spin. This final spin is not fast enough to ensure a robust enrichment of the final fraction with EVs and would have resulted in isolation of cellular debris as well as a larger proportion of apoptotic bodies compared to small EVs following such a high dose of radiation. For single vesicle analysis they used both NTA as well as TEM, although their included TEM image identifies significant cellular debris alongside their isolated EVs. Additionally, they performed flow cytometry using Annexin V fluorescent labelling, a protein which binds to phosphatidylserine a significant component of the EV lipid bilayer. However, there was no further assessment of positive or negative control markers.

With such a high dose of radiation, and the omission of a third faster centrifugation spin, the fraction used for subsequent injection into the rat model would likely have contained significant cellular debris in addition to the EV component. In fact, this can be seen in the TEM images which contains evidence of a non-EV component in their isolated fraction. Without further characterisation to assess for cellular debris, and repeat experiments using detergents and proteinases as a negative control, it is hard to conclude that the identified anti-tumour immunity is related to EVs and their cargo. The lack of functional interrogation and the minimal assessment of the mechanisms underlying these changes also limits the conclusions that can be drawn from these experiments.

Finally, by performing RNAseq on EVs isolated from a control and induced radioresistant GBM cells, Zhao et al. [21] were able to identify several circRNA and miRNA expression changes. They experimentally validated only their top hit, circATP884, using real time quantitative PCR to confirm increased levels in radioresistant cell-derived EVs. They subsequently co-cultured the isolated EVs with the control GBM cell line demonstrating transferrable radioresistance following one moderate dose single fraction radiation treatment.

They described a clear differential ultracentrifugation technique, utilising filtration prior to the final collection spin to try to further remove larger particles including cell debris, but it is unclear how long the media was conditioned prior to isolation. They employed NTA and TEM for single EV analysis, although their included TEM image demonstrates co-isolated debris as well as EVs. Additionally, the used fluorescent labelling to try to confirm EV uptake into recipient cells, albeit without a dye only control. However, there was no assessment of positive and control EV markers.

With the limitations in experimental design and reporting in this study, including a lack of clarity regarding media conditioning time and the absence of positive and control EV marker assessment, the ability to confidently allocate the changes in circRNA and miRNA identified directly to changes in EV content is difficult.

By comparing single fraction and multi-fraction radiation schedules, Baulch et al. [22] investigated whether fractionation schedule influenced radiotherapy-induced EVs effect recipient cell survival. They investigated several single fraction doses (1, 2 or 5 Gy) and multi-fraction doses (0.5 × 5, 1 × 5, 0.5 × 10, 1 × 10 and 0.5 × 20 Gy), and they were the only study to investigate multi-fraction regimes in cell lines. However, their single dose and multi-fraction regimes were not biologically equivalent. They demonstrated radioresistance following co-culture with both untreated control and multi-fraction radiation induced EVs, and induction of oxidative stress with single dose and multi-fraction radiotherapy. They also identified increases in recipient cell MMP2 levels but a decrease in MMP2 levels in the EVs themselves, suggesting the transfer of an MMP2 regulating factor.

They collected their conditioned media at a delayed 96-hours post-radiation timepoint and then isolated EVs using precipitation. They used NTA alone to characterise their EV fraction.

Despite their comprehensive radiotherapy protocol, albeit not biologically equivalent, there was minimal EV fraction characterisation reducing the confidence that the identified changes are due to alterations in EV cargo with radiation. Additionally, they used a significantly later post-radiotherapy timepoint for collecting conditioned media which may have affected their findings compared to other studies.

Mrowczynski et al. [23] identified an increase in EV release after radiation in a dose dependent manner (3 and 12 Gy). They also demonstrated increased radioresistance when co-culturing radiation-induced EVs with glioma cells, which was inhibited with simvastatin and heparin treatment (proposed EV uptake inhibitors), and they confirmed this using an in vivo mouse model. However, compared to Baulch et al. [22], they did not observe any changes in oxidative stress levels although they collected their conditioned media at different timepoints and used different radiation doses. Mechanistically, they identified increased oncogenic markers and decreased tumour suppressive markers, including upregulation of the NOTCH and Jak-STAT pathways, within the miRNA, mRNA, and protein cargo of their isolated EVs.

They used precipitation to isolate EVs, whilst their EV characterisation included both NTA and TEM for single EV analysis, although their TEM images demonstrate co-isolated biomolecules. They performed Western blots to assess EV markers but no assessment of control markers, and fluorescence uptake imaging to confirm EV uptake by recipient cells, albeit without dye only controls.

Whilst they have demonstrated the presence of EVs through their characterisation, and identified dose-response related changes, given the known risk of co-isolating active biomolecules with precipitation-based isolation methods then the lack of purity controls makes it difficult to conclusively associate the identified findings with changes in the EV cargo as opposed to changes in the secretome overall.

Finally, by comparing low dose (2 Gy) and high dose (10 Gy) radiotherapy doses, Wang et al. [24] identified preferential increase in circMETRN after low dose radiation. Co-culture of radiation-induced EVs with glioma cells led to increased proliferation and radioresistance, which was abrogated when circMETRN was inhibited using siRNA in the donor cell. Mechanistically, they identified that circMETRN sponged miR-4709-3p in recipient cells leading to increased GRB14 activity and increased PDGFRα expression level. They also describe increasing circMETRN levels in serum EVs during radiotherapy.

Whilst the cell culture experiments clearly described their whole protocol for isolating EVs by precipitation, there are no specific details on the processing of the patient serum prior to EV isolation. They performed both TEM and NTA as single EV analysis, identifying particles of the appropriate size and shape, alongside Western blot of EV and purity control markers.

This was a generally focussed and well performed study which clearly identified changes in circMETRN following low dose radiotherapy, which is clinically relevant for early radiation response or field edge effects. However, the lack of detail regarding the serum processing will impact on the clinical interpretation given that serum has a significantly increased platelet-derived EV component and different processing techniques can increase this component further through platelet activation(25). Whilst this doesn’t specifically detract from the findings of this study it does limit the ability to take this research forward as it is unclear if investigating platelet-derived EV circMETRN, tumour-derived EV circMETRN or other TME component-derived EV circMETRN would be most relevant.

### Radiotherapy-induced EVs effects on the TME

A total of six studies investigated the effects of radiotherapy induced EVs on various cells within the TME. Ultracentrifugation was used in four studies, whilst precipitation-based isolation was performed in two studies, and these will be discussed separately. All of these six studies investigated different components of the TME, with two studying neural cells, either astrocytes [26] or neural stem cells (NSC) [27], three investigating immune cells, investigating Natural Killer (NK) cells [28], T cells [29], or microglia [30], and the final study investigating the effects of radiotherapy-induced EVs on muscle cells [31].

Colangelo et al. [26] studied whether CD147 levels in GBM EVs was altered following radiation, and if that influenced astrocyte matrix metalloproteinase (MMP) secretion, a known TME-based mechanism by which tumour cells increase their invasiveness. They co-cultured an astrocyte cell line with EVs from three gamma-irradiated GBM cell lines, with a clear and comprehensive single fraction radiation protocol. They investigated different time points for conditioned media collection, demonstrating an increase in CD147 levels with radiation at 24 and 48 h but no difference in levels compared to the control by 72-hours in two of the three cell lines, and identified a dose dependent increase in EV CD147 in one of the cell lines. Tumour-derived EVs were taken up by astrocytes and led to increased MMP9 secretion through activation of the JNK pathway, but with no clear difference between the radiation and control experiments.

EV isolation was performed by differential ultracentrifugation. They used NTA and cryo-electron microscopy for single vesicle assessment, tested for positive and purity control markers by Western blot and included the Ponceau stain as a loading control. They performed sucrose density ultracentrifugation to confirm co-sedimentation of CD147 with a known EV marker (CD63) at the correct density for EVs, and performed fluorescent labelling to demonstrate EV uptake, albeit without a dye only control.

Despite a comprehensive research plan with appropriate EV techniques, the available evidence presented in this article demonstrates increasing CD147 levels following radiotherapy, and increasing astrocyte MMP secretion following GBM EV co-culture, but does not comprehensively link the increase in EV CD147 levels following radiation to changes in astrocyte MMP secretion.

Tian et al. [29] investigated the interaction between radiotherapy-induced GBM-derived EVs and T-cells using co-culture. The radiotherapy details are not described in the methods, however, from the information identified in the figures, they performed a dose-response assessment using doses of 0, 2, 4, and 8 Gy. They identified increases in FoxP3 + regulatory T cells and a reduction in Th1 cells in a dose-dependent manner, which they subsequently confirmed in a mouse subcutaneous tumour xenograft model. They performed extensive mechanistic studies identifying increased levels of B7-H4 in radiotherapy induced EVs was due to increased interactions between ALIX (a key EV biogenesis enzyme) and ATM (a keystone DNA damage response pathway kinase). This increase in EV B7-H4 led to reduced STAT1 pathway phosphorylation which increased FoxP3 expression and impaired Th1 differentiation. Finally, inhibiting the packaging of B7-H4 into EVs by GBM cells, using GW4869 or B7-H4 shRNA treatment, led to reduced tumour growth and longer survival following irradiation in their in vivo mouse model, with radiation-induced increased B7-H4 leading to radioresistance.

They performed ultracentrifugation to isolate EVs, albeit without full description of the length of their spins. Subsequent characterisation involved NTA of the whole fraction, plus Western blot analysis of known EV and purity control markers. Iodixanol density gradient centrifugation was used to confirm co-isolation of B7-H4 with EV markers (ALIX and Rab7).

This was a well performed and comprehensive study, which incorporated enough characterisation of the isolated EVs with upstream and downstream investigation of the identified biological effects to link the findings to changes in the EVs cargo following radiation.

By studying microglia co-cultured with irradiated glioma cells (2 Gy), Zhang et al. [30] identified reduced phagocytic activity and the promotion of microglial polarisation to a pro-tumourigenic M2 phenotype subsequently leading to increased GBM proliferation. This effect was inhibited by GW4869 treatment and replicated with EV co-culture. Additionally, they identified increased EV secretion following radiotherapy. They next demonstrated circRNA changes, including circ_0012381 increases, which sponges with miR-340-5p resulting in M2 microglial polarisation. They identified that mir-340-5p targeted ARG1 in the absence of radiotherapy-induced EV uptake, and that C/EBPB transcriptionally regulates the expression of circ_0012381.

They used ultracentrifugation protocol to isolate EVs, but it is unclear how long the media was conditioned for prior to collection. They used both NTA plus scanning electron microscopy for single EV analysis, however there was no assessment of possible co-isolated contaminants.

Whilst there was extensive signalling characterisation to understand how the identified circRNA changes led to different microglia polarisation states, the minimal EV characterisation makes it difficult to confidently assigned these changes to transfer of EV cargo.

To investigate glioma induced cachexia, Shin et al. [31] performed co-culture of radiotherapy-induced EVs (6 Gy) with a myoblast cell line and a differentiated myotube cell line. They showed that this co-culture led to muscle atrophy, associated elevations in cachexia markers, activation of the mTOR/AKT pathway in a time dependent manner, and activation of the STAT3 signalling pathway through EV transfer of PA1. They confirmed increased PA1 protein, not mRNA levels, in the recipient cell to confirm that there was a transfer of PAI1 protein from irradiated GBM cells to myoblast and myotube cells.

They performed differential ultracentrifugation, including filtration before their final EV collection spin, to isolate their EVs. Subsequently, they used TEM for single vesicle analysis, Western blot of only EV markers, and DiO fluorescent tagging to confirm EV uptake, albeit without a dye only control.

This study was not specifically focussed on EVs, instead looking to understand their finding of transfer of PAI-1 from GBM cells to muscle cells following radiation. Whilst they demonstrated the presence of EVs prior to co-culture, it is difficult to confidently link the transfer of PAI-1 to an EV mediated mechanism due to the lack of purity control markers in their Western blots and without protease/detergent experiments which would have strengthened the evidence that EV cargo transfer is responsible for the recipient cell phenotype.

Through co-culturing NSC with irradiated glioma cells or glioma cell-derived EVs, Yang et al. [27] sought to investigate radiation induced bystander effects on memory. They demonstrated that NSC proliferation and differentiation were inhibited in both cell and EV co-culture following one high single fraction radiation dose (10 Gy). EV fraction injection into an in vivo mouse model led to neurocognitive degeneration in some of the investigated assessments as well as impaired neurogenesis.

They used only 3 h of media conditioning prior to isolating EVs using a precipitation kit. Confirmation of EV isolation was by NTA alone although their NTA protocol is not clearly described in the methods. However, EV uptake by NSC was investigated using fluorescent labelling, albeit without a dye only control.

With the limited characterisation and use of a precipitation-based isolation method it is difficult to confirm these effects to EVs as opposed to a co-separated biomolecule. Additionally, given that the radiotherapy dose used is not a clinically relevant dose and the short timescale after radiotherapy that the conditioned media was collected it is unclear whether these findings are directly clinically relevant.

Investigating the effect of radiotherapy-induced EVs on NK cells, Briand et al. [28] identified that following a high single fraction dose of radiation (10 Gy) GBM cell TET2 mediated demethylation of the miR-378 promoter led to increased EV miR-378a-3p, reducing the cytotoxicity of NK cells by decreasing their granzyme B levels. They demonstrated that circulating blood miR-378-3p levels correlated with circulating granzyme B levels in two GBM patients.

They utilised precipitation to isolate EVs, plus a commercial kit to isolate miRNA from EVs directly from blood. There is no description of characterisation, and minimal detail regarding cell line irradiation. They used GW4869 to investigate whether their findings were due to EVs, although given that GW4869 works to reduce small EV production by increasing larger EV production [32], without EV characterisation it is difficult to confirm this assessment.

This was a short communication which clearly limited the detail that could be included in the publication. However, this lack of detail and absence of clear EV characterisation makes it difficult to assess whether the findings are due to changes in EVs.

### Studies investigating the effect of EVs on radiation response

Five studies investigated the effect of EVs on GBM cell line radiosensitivity, of which two used precipitation-based isolation methods and three performed differential ultracentrifugation. Two studies investigated GSC-derived EVs, one study investigated macrophage-derived EVs, one investigated the effect of different levels of AHIF, the natural antisense transcript of hypoxia inducible factor-1α [33], and one study investigated the role of hypoxia by directly culturing cells in a hypoxic environment [34].

**Table 2:** A summary of studies investigating the effect of EVs on radiation response in GBM

Author	Year	Donor cell	EV isolation	EV confirmation	Recipient cells	Radiotherapy details	Results
Dai [33]	2019	4 GBM cell lines (U87, U251, A172, T98G) with wild type, overexpression, or knockdown of AHIF.	48-hour conditioned media. Centrifugation (1000 g 10 min, 3000 g 30 min) followed by Total Exosome Isolation Reagent.	Transmission electron microscopy to assess size. Western blot of EV (CD63 and CD81) and purity control (Cox IV) markers. Recipient cell uptake using PKH67 fluorescent labelling. Repeat experiment using GW4869 (EV secretion inhibitor).	4 GBM cell lines (U87, U251, A172, T98G).	Modality: Caesium gamma irradiation. Treatment: 0 and 6 Gy in 1 fraction.	AHIF induced radioresistance following radiation is transferrable via EVs.
Yue [34]	2019	3 GBM cell lines (U87, LN229, U251) incubated at 5% or 95% CO2. 32 patients (12 GBM).	?hour conditioned media. Centrifugation (3000 g 15 min) with 0.45 μm filtering followed by ExoQuick precipitation kit.	No clear description of confirmation in methods. Quantification of nanoparticle tracking analysis and Western blot for EV markers (CD9, CD63, CD81, HSP70) on donor cell lysates in figures.	3 GBM cell lines (U87, LN229, U251) incubated at 5% CO2.	Modality: Cabinet xray irradiation. Treatment: 0, 2, 4, 6 and 8 Gy in 1 fraction in cells. 0 and 40 Gy in 5 fractions in mice.	Hypoxia-induced miR-301a can be transferred to induce radioresistance through upregulation of wnt/ β-catenin pathway.
Zhang [37]	2020	Healthy volunteer monocytes differentiated to macrophages.	48-hour conditioned media. Differential ultracentrifugation: 300 g 10 min, 2000 g 10 min, 10,000 g 30 min with 0.22 μm filtering after each spin, 100,000 g 70 min, washed with PBS and 100,000 g 70 min.	Transmission electron microscopy and qNANO analysis to assess size and concentration. Western blot of EV (CD9 and TSG101) and purity control (calnexin) markers. Recipient cell uptake using PKH67 fluorescent labelling. Repeat experiment using EV depleted condition media.	3 different glioma stem cell lines (G8-11, G20, G267) + 1 glioma stem cell xenograft mouse model (G8-11).	Modality: Electron irradiation via linear accelerator. Treatment: 0 and 6 Gy in 1 fraction for cells. 0 and 10 Gy in 4 fractions for mice.	Increased glioma stem cell growth, upregulation of mesenchymal subtype markers and increased radioresistance following co-culture with M2 polarised macrophages.
Ma [35]	2022	Fresh glioma stem cell lines from 3 patients.	Twice weekly harvested (? hour) conditioned media. Differential ultracentrifugation: 300 g 5 min, 2000 g 10 min, 10,000 g 30 min, 100,000 g 70 min, washed with PBS and 100,000 g 70 min.	Cryo-electron microscopy and Nanosight NS300 analysis to assess size and concentration. Western blot of EV markers only (Alix, TSG101, CD81, CD9). Recipient cell uptake using DiI fluorescent labelling.	2 GBM cell lines (LN229, U118).	Modality: Cobalt gamma irradiation. Treatment: 2 and 5 Gy in 1 fraction for cells.	Glioma stem cell-derived EVs increase the radioresistance, migration and invasion of recipient glioma cells.
Panizza [36]	2023	8 primary GSC cultures and 1 GBM cell line (U-87MG).	48-hour conditioned media. Ultracentrifugation: 100 g 5 min, 100 g 5 min, 0.22 μm filtering (to collect microvesicles), 100,000 g 180 min (to collect exosomes).	Transmission electron microscopy and Nanoparticle tracking analysis using NS300 to assess size and concentration. Proteomics to demonstrate increased EV markers and depleted purity control markers. Repeat experiments using microvesicle depleted conditioned media, intermittent co-culture (4 days co-cultured, followed by 4 days not co-cultured) and treatment with non-vesicle-extracellular protein.	1 mouse fibroblast cell line (NIH/3T3).	Modality: Caesium gamma irradiation. Treatment: 6 Gy	NAMPT-high radioresistant GSC-derived microvesicles increase the radioresistance of recipient glioma cells through transfer of NAMPT.

Ma et al. [35] demonstrated that, following irradiation, co-culture with three primary GSC cell line-derived EVs increases radioresistance, migration and invasion of recipient glioma cells. Subsequent miRNA profiling of the isolated EVs identified 25 miRNA consistently elevated in each of the three GSC cell lines, of which eight were predicted to target the PTEN pathway.

EV isolation was by differential ultracentrifugation from GSC-conditioned media harvested twice weekly. EV characterisation was performed by cryo-electron microscopy and NTA to identify particles of the correct size and shape, plus Western blot for several EV markers, but not co-isolating contaminants. They demonstrated EV uptake by recipient cells through fluorescent (DiI) tagging, although without a dye only control.

This was a generally suitable protocol with a clearly described characterisation process, however the lack of assessment of co-isolated biomolecules reduces the confidence that the identified changes are due to EVs and their cargo as opposed to co-isolated biomolecules.

Investigating primary radioresistant primary GSC’s Panizza et al. [36] demonstrated that radioresistant GSC-derived microvesicle mediated transfer of NAMPT led to radioresistance of recipient cells. However, most of the experiments were performed in murine fibroblast cell lines, with only limited confirmation in recipient GSC lines.

They used a 0.22 μm PVDF filter to collect “microvesicles” followed by ultracentrifugation of the supernatant to isolate “exosomes”. They used TEM and NTA for single vesicle analysis, followed by analysis of the proteomics from the “exosome” and “microvesicle” samples to demonstrate enrichment of known EV markers and depletion of cellular markers compared to parental cell lysate. They also performed several functional validation experiments including the use of “microvesicle” depleted conditioned media and treatment with non-vesicle extracellular NAMPT to demonstrate the need for NAMPT within the cargo of an EV, and intermittent co-culture experiments whereby they treated recipient cells with EVs for 4 days then without EVs for 4 days to demonstrate the need for continued EV exposure.

Despite an unusual EV isolation protocol and the limited confirmation when using recipient GSC lines, the characterisation protocol and robust functional interrogation of their findings gives strong evidence that transfer of NAMPT in EVs provides transferrable radioresistance in glioma.

Through co-culturing GSC with with pro-tumourigenic M2 polarised macrophage-derived EVs, Zhang et al. [37] demonstrated increased GSC growth, upregulation of mesenchymal subtype markers, and increased radioresistance compared to co-culture with non-polarised M0 macrophage-derived EVs. They subsequently confirmed these findings using an in vivo xenograft model. miRNA sequencing of M2 macrophage-derived EVs identified miR-27a-3p, miR-22-3p and miR-221-3p as regulators of this effect through interactions with CHD7.

They used differential ultracentrifugation for EV isolation followed by a robust EV fraction characterisation. They used TEM and tunable resistive pulse sensing (another suitable technique) for single EV analysis plus western blots for EV markers and a purity control marker. They demonstrated EV uptake using fluorescent tagging (PKH67), albeit without a dye-only control, and additionally they co-cultured GSC with conditioned media, EV depleted conditioned media, and EVs to confirm that the isolated EV component was required for their identified effects.

This was a comprehensive and robust methodology with repeat functional studies to confirm the necessity of the isolated EV fraction. However, their TEM image demonstrates some co-isolated biomolecules and their functional studies do not conclusively exclude the possibility that a co-isolated biomolecule may induce the identified effects.

After demonstrating upregulated AHIF levels in GBM cells following irradiation (6 Gy) using real time quantitative PCR, Dai et al. [33] identified that AHIF levels were inversely correlated with radiosensitivity using lentiviral AHIF overexpression and knock down experiments, and that co-culture of EVs from cells with over-expression or knockdown of AHIF could transfer AHIF-mediated radiosensitivity.

They used a precipitation-based kit for EV isolation. They characterise their isolated fraction reasonably robustly using TEM and Western blot using known EV markers and an intracellular contaminant marker (COX IV a mitochondrial protein). Additionally, they perform fluorescent uptake, without a dye only control, and EV secretion inhibition (using GW4869) to try and demonstrate that their findings were related to EV transfer between donor and recipient cells.

Despite the clear EV isolation and characterisation protocol described in this publication, there are some challenges in assessing these results due to some inconsistencies in the included figures, the accumulation of co-isolated biomolecules as demonstrated in their TEM figure, and the lack of functional controls such as detergent/protease experiments.

Yue et al. [34] investigated the transfer of hypoxia-induced miR-301a by EVs and demonstrated increasing radioresistance through activation of the wnt/β-catenin pathway.

They first filtered their conditioned media through a 0.45 μm filter to remove large debris, following which they used a precipitation kit to isolate EVs. There are limited methodological details, and it is unclear how long they conditioned their media for before analysis which would affect the EV concentration within the media. There is no detail regarding their EV confirmation protocol described in their methods. Despite this omission, they include figures demonstrating EV characterisation using NTA, plus Western blot images of known EV markers but only on the lysate of the donor cells which they used to demonstrate increasing EV marker concentrations under hypoxic conditions.

There is a lack of detail regarding the EV methodology included in this paper, with minimal characterisation, which severely limits the conclusions regarding the role of EVs in their finding of hypoxia-inducible transferrable radioresistance.

#### Clinical studies

Five studies investigated EV changes with radiotherapy for GBM patients.

**Table 3:** A summary of studies investigating changes in EVs during or following radiotherapy in GBM patients

Author	Year	Number of patients	Sample timings	Sample	EV isolation	EV confirmation	Results
Reynés [38]	2013	22 GBM patients 40 volunteers	One sample pre-radiotherapy, and one during final week of radiotherapy.	Sodium citrate tubes.	First 3ml blood discarded. Plasma isolated from blood (1500 g for 30 min).	Flow cytometry of EV marker only (Annexin V)	Higher EV counts in patients vs. controls. EV counts reduced with treatment. No correlation between EV counts (pre- or post-) and overall survival.
Koch [39]	2014	11 GBM patients 7 volunteers	One sample pre-radiotherapy, one post-radiotherapy, and 1, 3-, 6-, 12- and 24-months post-radiotherapy. Volunteers had 1–2 samples.	2.8ml in sodium citrate tubes.	Plasma isolated from blood (300 g 20 min), and centrifuged (2500 g 20 min) to remove platelets. Platelet-free plasma centrifuged (15,000 g 30 min).	Cryo-electron microscopy to assess size. Flow cytometry of EV markers only (Annexin V, EGFR).	EV counts were higher in patients with progression vs. pseudo-progression.
Evans [40]	2016	16 GBM patients	One sample pre-radiotherapy, twice during, and one post- radiotherapy.	2.8ml in sodium citrate tubes.	Plasma isolated from blood (300 g 20 min), and centrifuged (2500 g 20 min) to remove platelets. Platelet-free plasma centrifuged (15,000 g 30 min).	Cryo-electron microscopy to assess size. Flow cytometry of EV (Annexin V, EGFR) and purity control (CD41 and CD235) markers.	Increasing EV counts during radiotherapy was associated with recurrence and death.
Li [41]	2020	?5 GBM patients	One sample pre-radiotherapy and one post-radiotherapy.	EDTA tubes.	Whole blood stored at -4 then centrifuged (12,000 g 10 min) to isolate plasma. Plasma incubated with ExoQuick precipitation kit.	Electron microscopy (not shown in paper) and nanoparticle tracking analysis to assess size and concentration. Western blot for EV markers only (CD9, HSC70, TSG101).	Changes in metabolic pathway miRNA expression in EV cargo were identified following radiotherapy.
Tzaridis [42]	2021	67 GBM patients (36 + 31 in independent cohorts) 22 volunteers	One sample pre-radiotherapy (28 patients) and at 2–4 surveillance MRI visits.	9ml in serum tubes.	Serum isolated from blood (2000 g 15 min), centrifuged (3200 g 20 min) to remove platelets, and filtered 0.45 μm. Size exclusion chromatography (qEV column, IZON) used with 0.5ml serum. Fractions 8–10 pooled and concentrated using ultracentrifugation (110,000 g 105 min).	Transmission electroon microscopy and ZetaView nanoparticle tracking analysis to assess size and concentration. Western blot of EV (CD9, Flotillin-1) and purity control (Calnexin, GAPDH, Apo-A1) markers. Flow cytometry of EV (CD9, CD63, CD81) and investigational surface markers.	Higher EV counts in patients vs. controls. No change in EV counts longitudinally, and not correlated with extent of resection. Identified 6 surface markers upregulated in GBM, with 3 upregulated at progression.

Firstly, Reynés et al. [38] compared differences in phosphatidylserine positive EVs (a large EV surface marker) between 22 GBM patients, prior to and in the final week of radiotherapy, and 40 healthy volunteers. They identified significantly increased EV levels in patients with GBM compared to volunteers, and that EV concentration reduced with radiotherapy. However, they found no association between EV counts (pre- or post-radiotherapy) with overall survival.

They performed flow cytometry using a fluorescently labelled antibody against Annexin V (which targets phosphatidylserine) to quantify phosphatidylserine positive EVs, however there was no further attempt to characterise the EV population.

The findings of differences in EV concentration between GBM patients and healthy volunteers, as well as changes in EV concentration with treatment, raise the possibility that EVs could be used as a liquid biopsy biomarker. The lack of characterisation of EVs is a clear limitation but not unexpected given it is a very early study, which was published prior to the release of any of the ISEV guidelines.

Koch et al. [39] and Evans et al. [40] reported an overlapping population of 11/16 GBM patients respectively. Koch et al. reported [39] that phosphatidylserine positive EV counts were higher with imaging-defined progression. Subsequently, Evans et al. [40] identified that increasing phosphatidylserine positive EVs during radiotherapy was associated with increased recurrence and death.

They utilised Annexin V targeted flow cytometry-based immunocapture to quantify EVs from platelet depleted plasma. Subsequent EV characterisation used cryo-electron microscopy on plasma prior to EV isolation for both publications, whilst Evans et al. [40] also performed flow cytometry of negative markers (CD41 and CD235).

This is a reasonable and reproducible isolation method focussed primarily on quantifying changes in EVs, and so despite the limited characterisation, the results are reasonably robust. These are interesting and hypothesis generating findings, that EVs could be used as prognostic or monitoring biomarkers, however given the small number of patients included, larger validation studies are required.

Investigating miRNA changes within EV cargo following radiotherapy, Li et al. [41] identified several metabolic pathway miRNA changes. It is not clearly documented; however, it is likely that they investigated EVs from 5 GBM patients.

They used kit-based precipitation to isolate EVs following an unusual plasma isolation technique. For characterisation, in their results they mentioned NTA, and electron microscopy to visualise EVs, plus Western blot for EV markers, but not co-isolated contaminants. However, there was no NTA or Western blot protocol in their methods, and no description or figure for electron microscopy.

Given the known risk of co-isolating various bioactive molecules following precipitation-based isolation methods and the extremely limited characterisation process, it is challenging to link the identified changes specifically to EV cargo changes as opposed to general secretome changes.

Finally, studying two independent GBM cohorts for a total of 67 GBM patients and 22 healthy volunteers, Tzaridis et al.(42) investigated changes in serum EVs as a possible blood based liquid biopsy. They identified significantly greater number of EVs in the GBM patients’ serum compared to healthy volunteer serum, albeit with significant interpatient variation. There was no significant change in EV count overtime including at the time of progression, and no correlation between EV counts and extent of surgery. In their novel biomarker investigations, they identified six upregulated surface markers (CD29, CD44, CD81, CD146, C1QA and Histone H3), of which three (C1QA, CD44 and Histone H3) were upregulated in patients with progressive disease compared to stable disease.

They used size exclusion chromatography to isolate EVs from 0.5ml serum, followed by ultracentrifugation to concentrate their isolated EV sample. They characterised their EV sample using TEM and NTA for single vesicle analysis, performed Western blots of known EV markers and multiple purity control markers including an assessment of Apolipoprotein A1 contamination, a frequently co-isolated biomolecule when using size exclusion chromatography. Additionally, as part of their investigations they performed flow cytometry of three known EV surface markers as well as multiple investigational surface markers.

This was an extremely well performed study, and as the most recently published clinical study it also has the most comprehensive characterisation of all clinical studies. In agreement with previous studies, they identified significantly increased EV counts in GBM patients, however they found no clear association between EV counts and tumour volume, either extent of resection or progressive disease, a different finding to other groups [38–40].

## Discussion

Through systematically reviewing the literature we have been able to comprehensively describe the range and extent of isolation and characterisation techniques used to investigate EV-radiotherapy interactions in GBM and how this influences the conclusions drawn from these studies.

We can see that the number of studies investigating EV-radiotherapy interactions in GBM has significantly increased from 6 (3x immunocapture, 2x precipitation, and 1x differential ultracentrifugation) to 20 (1x immunocapture, 1x size exclusion chromatography, 7x precipitation and 11x differential ultracentrifugation) following MISEV2018 [12] (Table 4).

**Table 4:** A summary of studies by year highlighting the increase in studies and changing isolation methodology

Author	Year	Era	Subcategory		EV isolation
Tzaridis [42]	2023	Post-MISEV2018	Clinical study	Clinical	Size exclusion chromatography
Panizza [36]	2023		Effect of EVs on radiosensitivity	Glioma	Differential ultracentrifugation
Whitehead [17]	2023		Effect of radiotherapy on EVs	RT only	Differential ultracentrifugation
Jennrich [14]	2022		Effect of radiotherapy on EVs	RT only	Immunocapture
Ma [35]	2022		Effect of EVs on radiosensitivity	Glioma	Differential ultracentrifugation
Zhang [31]	2022		Effect of radiotherapy on EVs	Immune	Differential ultracentrifugation
Tian [30]	2022		Effect of radiotherapy on EVs	Immune	Differential ultracentrifugation
Shin [32]	2022		Effect of radiotherapy on EVs	Other	Differential ultracentrifugation
Yang [27]	2021		Effect of radiotherapy on EVs	Neural	Precipitation
Wang [24]	2021		Effect of radiotherapy on EVs	Glioma	Precipitation
Briand [28]	2020		Effect of radiotherapy on EVs	Immune	Precipitation
Li [41]	2020		Clinical study	Clinical	Precipitation
Ramakrishnan [15]	2020		Effect of radiotherapy on EVs	RT only	Precipitation
Colangelo [26]	2020		Effect of radiotherapy on EVs	Neural	Differential ultracentrifugation
Zhang [37]	2020		Effect of EVs on radiosensitivity	Immune	Differential ultracentrifugation
Dai [33]	2019		Effect of EVs on radiosensitivity	AHIF	Precipitation
Yue [34]	2019		Effect of EVs on radiosensitivity	Hypoxia	Precipitation
Pavlyukov [19]	2019		Effect of radiotherapy on EVs	Glioma	Differential ultracentrifugation
Pineda [20]	2019		Effect of radiotherapy on EVs	Glioma	Differential ultracentrifugation
Zhao [21]	2019		Effect of radiotherapy on EVs	Glioma	Differential ultracentrifugation
Mrowczynski [23]	2018	Pre-MISEV 2018	Effect of radiotherapy on EVs	Glioma	Precipitation
Baulch [22]	2016		Effect of radiotherapy on EVs	Glioma	Precipitation
Evans [40]	2016		Clinical study	Clinical	Immunocapture
Koch [39]	2014		Clinical study	Clinical	Immunocapture
Reynés [38]	2013		Clinical study	Clinical	Immunocapture
Arscott [18]	2013		Effect of radiotherapy on EVs	Glioma	Differential ultracentrifugation

Despite this increase, there remains a lack of research investigating EV radiation interactions, with only 145 publications including “radiotherapy OR radiation” plus “extracellular vesicles” compared to 4539 including “extracellular vesicles” alone identified via Pubmed as published in 2022. As highlighted below, extracellular vesicles could increase our understanding of several areas of current uncertainty in radiobiology, and so we would encourage collaboration between EV researchers and radiobiologists/radiation oncologists.

### The impact on local tumour control

The primary aim of radiotherapy is to provide tumour control through tumour cell killing. However, there is clear evidence that secondary tumour radioresistance can develop following radiation either directly through tumour cell to tumour cell communication [43] or indirectly through the further development of a tumour permissive TME [44]. Mechanistically, EVs could be involved in facilitating the development of radioresistance by both direct and indirect pathways.

Indeed, despite the divergent aims of these diverse studies incorporating varied methodologies and different targets of interest, there is evidence for both pathways in the studies included in this review. In fact, the main overarching common theme of these studies is the induction of transferable radioresistance to recipient glioma cells. This clearly highlights the need to better understand the communication between cells within the GBM TME in response to radiation to enable a more complete model of radiation response and radioresistance to be developed. However, given that EV transfer is multi-directional, there are a lack of studies investigating the interactions between multiple components within the complex GBM TME [45] following radiation. Understanding the effect of radiation on the production of EVs from multiple cell types will add significant complexity to the experimental set up, as trying to identify the effects from EVs of individual cell types within a complex multi-cellular co-culture is currently beyond the limit of our current experimental techniques. However, it is this level of complexity which will ultimately provide the most relevant answers. A systematic comparison of radiation effects within the GBM TME should preferably also catalogue changes in multiple different bioactive molecules including protein and RNA subtypes to provide a reference for comparison with future pre-clinical and clinical studies.

The role of EVs in radioresistance should also be incorporated into the traditional radiobiological understanding of radiation response. Classical radiobiology identified the 5 ‘R’s: DNA Repair, cell cycle Redistribution, Reoxygenation, tumour Repopulation, and intrinsic Radiosensitivity [46], which modulate the cellular response to radiation. Most of the research in this review has investigated changes in intrinsic radiosensitivity, however one study [34] investigated the role of hypoxia in modulating EV signalling were identified. Despite the significant methodological limitations of this study as discussed above, this is an extremely important hypothesis generating concept. The possibility that hypoxia-induced radioresistance is transferrable between cells through EV-mediated mechanisms raises the possibility that even small volumes of hypoxic clones within the tumour may give rise to hypoxia induced radioresistance. This finding therefore needs further investigation using robust and up-to-date EV methodology. Additionally, there is emerging consensus that hypoxia is not a binary state within tumours, with evidence for chronic, acute, and cycling hypoxia existing within tumours [47]. The study identified in this systematic review [34] performed their experiments using stable whole culture chronic hypoxia, and so additional future work using more physiological hypoxia is required.

### The impact on late effects

In addition to its effect on the tumour, radiotherapy can lead to significant and irreversible late effects [48]; indeed, the choice of radiation dose is a balance between increasing tumour control and minimising these late effects. Over the years, there has been significant improvements in our knowledge of organs tolerances to different doses of radiation [49], and yet our understanding of the underlying mechanism leading to the development or radiotherapy late effects is more limited [50]. The studies by Yang et al. [27] and Shin et al. [31] sought to understand how radiotherapy induced-EVs might mechanistically impact cognition and cachexia respectively. They provide a novel possible explanation for the stochastic effects of radiation, whereby the development of a toxicity is unrelated to the dose of radiation to a particular structure. Whilst limited detail regarding specific mechanisms can be drawn from these studies, they do provide a new framework for thinking and investigating radiotherapy toxicities. This framework could easily be expanded to facilitate a comprehensive investigation of the mechanistic causes of radiotherapy late effects in GBM through the assessment of functional changes in the desired organ at risk following co-culture of target organ cells with EVs derived from both GBM cells and the desired target organ cells treated with a systematic range of single dose and fractionated doses of radiotherapy.

### The impact on the immune environment

Despite overwhelming evidence that radiation alters the immune environment, and that the immune system affects the response to radiotherapy, currently there are only four studies investigating the EV interplay between GBM and immune cells following radiation, with each study investigating different immune cells. M2 polarised tumour associated macrophages and tumour associated microglia are the dominant immune cells within the GBM TME [51] and have been shown to exert a dominant immunosuppressive and tumour permissive niche [52]. The studies by Zhang et al. [30] and Zhang et al. [37] both suggest that following radiation, pro-tumourigenic M2 polarised tumour associated macrophages/microglia can lead to radioresistance and promote tumour growth, with some evidence that radiation can also promote the polarisation to M2 phenotype in these cells. In summary, these articles all provide evidence, of varying quality, suggesting that a dynamic communication between GBM cells release and immune cells occurs and results in a feedback loop driving an immunosuppressive phenotype within the TME which in turn leads to a tumour permissive and radioresistant tumour niche within the TME.

The awareness that several competing interactions occur between immunotherapy and radiotherapy [53–56], has led to several approaches attempting to combine immune checkpoint inhibitors with radiotherapy in multiple tumour types [57–60]. However, this approach has repeatedly failed in GBM with several negative large Phase 3 clinical trials [61–63]. With the finding identified in this review implicating EVs as a potential mechanism driving the immune-suppressive TME and a possible direct cause of radioresistance, understanding these interactions further must therefore be a priority to facilitate an effective use of immunotherapeutics in GBM, especially in combination with radiotherapy.

### Potential as a liquid biopsy

The need for a liquid biopsy for GBM is of paramount importance(64) given the difficulties in performing repeated tumour biopsies and the limitations in current imaging techniques(10). Several of the included studies demonstrate increasing EV concentrations following radiotherapy, whilst Zhao et al.(21) demonstrated clear differences in the EV cargo of control and radioresistant GBM cell lines. This enrichment in tumour derived-EVs and the ability to identify developing radioresistance during radiotherapy would be extremely valuable as a liquid biopsy. Yet, there are discordant findings between studies, for example there are some findings which suggest that EV concentrations track with tumour bulk(38–40), whilst some find no association(42). These are clearly interesting pilot data which highlight that peripheral monitoring of EVs could inform on patients’ clinical status during/following radiotherapy; however, they lack the power to clearly demonstrate EVs utility as a liquid biopsy at the present time. Additionally, the lack of rigour in EV characterisation and the minimal biological interrogation in the clinical studies limits our understanding of the biological processes occurring after radiotherapy in the clinical setting.

### Strengths and limitations

Tumour type specific differences exist regarding composition of the TME [65] and radiation response [66], and so understanding the interaction between EVs and radiation will also have tumour type differences. This is the first review to systematically investigate the published literature regarding radiotherapy and EV interactions within the context of GBM, although this, by necessity, reduced the studies eligible for inclusion in this systematic review.

However, including every study investigating radiotherapy-EV interactions in GBM results in a broad and scattered pool of publications to compare despite this common theme. This heterogeneity between the identified studies is compounded by the lack of consistency in marker analysis, and so this limits the ability to pull together a coherent biological narrative from the current evidence. Clearly there are multiple significant proteomic and RNA changes following radiotherapy, however the downstream effects and direct link to EVs is challenged by the frequent lack of EV characterisation and validation.

Further, despite a generous and inclusive search strategy, the requirement for publication in English and the use of Pubmed and Web of Science alone may have resulted in the omission of some relevant papers. Despite this limitation, the conclusions of this review are unlikely to be altered by the inclusion of any non-identified papers. Specifically, that there are increasing numbers of relevant publications in this topic albeit still few absolute publications, and the need for expertise in EV research to plan appropriate and robust experiments.

## Conclusions

EVs are an extremely valuable tool for understanding TME intracellular communication and variations in tissue level and interpersonal radiosensitivity. However, standardisation and robust characterisation is required to conclude that identified effects are due to EVs and their cargo. Through systematically reviewing the literature we identified frequent gaps in verification from published studies resulting in a significant risk of bias. We would encourage less EV-focussed researchers to collaborate with more experienced groups to ensure the robustness of their research findings.

## Data Availability

All data generated or analysed during this study are included in this published article.

## Abbreviations

EVs: Extracellular Vesicles
TME: Tumour microenvironment
GBM: Glioblastoma
MISEV2018: Minimal information for studies of extracellular vesicles 2018
PRISMA: Preferred reporting items for systematic reviews and meta-analyses
TEM: Transmission electron microscopy
NTA: Nanoparticle tracking analysis
GSC: Glioma stem cell
NSC: Neural stem cell
